# Dietary Acid Load, IGF-1 Single Nucleotide Polymorphism and Bone Resorption among Postmenopausal Chinese Women

**DOI:** 10.3390/nu10070915

**Published:** 2018-07-17

**Authors:** Sook Yee Lim, Mohd Shariff Zalilah, Yit Siew Chin, Vasudevan Ramachandran, Yoke Mun Chan

**Affiliations:** 1 Department of Nutrition and Dietetics, Faculty of Medicine and Health Sciences, Universiti Putra Malaysia (UPM), Serdang 43400, Malaysia; l.sookyee@yahoo.com (S.Y.L.); zalilahms@upm.edu.my (M.S.Z.); chinys@upm.edu.my (Y.S.C.); 2 Research Center of Non-communicable Diseases, Faculty of Medicine and Health Sciences, Universiti Putra Malaysia (UPM), Serdang 43400, Malaysia; 3 Malaysian Research Institute on Ageing, Universiti Putra Malaysia, Serdang 43400, Malaysia; vasudevan@upm.edu.my

**Keywords:** acid-base homeostasis, potential renal acid load, rs35767, diet, bone

## Abstract

The interaction of dietary and genetic factors may affect the development of bone deterioration. This study investigated whether the effects of dietary acid load (DAL) on bone loss in postmenopausal Chinese women were moderated by the insulin-like growth factor-1 (IGF-1) single nucleotide polymorphism, a known gene that plays a role in the regulation of bone formation and bone remodeling. A total of 217 healthy participants were recruited from the National Council of Senior Citizens Organizations Malaysia. Serum collagen type 1 cross-linked C-telopeptide was used as a surrogate bone marker to assess bone resorption and Agena^®^ MassARRAY genotyping analysis was used to identify the signaling of IGF-1 rs35767. The dietary acid load was measured by potential renal acid load score while physical activity was ascertained using the Global Physical Activity Questionnaire. Hierarchical regression was applied to test the main and interaction effects of DAL and IGF-1 genotypes in bone resorption. The result supported the diet-dependent acid-base balance theory that higher DAL was positively associated with bone resorption (β = 0.152, *p* = 0.031, *F*_(6,207)_ = 2.11, sig-*F* = 0.036, *R*^2^ = 0.079). However, the results indicated that there was no significant correlation between IGF-1 and bone resorption, or any significant interaction between DAL and IGF-1. In conclusion, there was no moderating effect of IGF-1 on the relationship between DAL and bone resorption.

## 1. Introduction

Osteoporosis is a skeletal disorder characterized by a low bone mass and bone microarchitectural deterioration which represents a major worldwide healthcare problem that affects more than 200 million people [1]. Hip fractures are the most serious outcome of osteoporosis while vertebral fractures are more common and have a substantial impact on mortality and morbidity [2]. The causes of osteoporosis and bone fractures are multifactorial and include lower vitamin D, aging, being women, low calcium intake, physical inactivity, and estrogen deficiency [3,4]. Women are more susceptible to osteoporosis than men with 80% of osteoporosis patients being women, especially postmenopausal women [3,5]. During the post-menopausal period, the sharp decline in production of estrogen may reduce the rate of bone formation and increase bone turnover [5]. Based on the aging trend of world’s population, 50% of all hip fractures would occur in Asia by 2050 [6]. In a multi-ethnicity country like Malaysia, Chinese women were reported to have the highest prevalence of hip fracture of all ethnicities [7].

In light of bone deterioration leading to bone fragility and bone fracture risk as well as deterioration of life and higher mortality risk, maintenance of bone health is a public health priority. Dietary factors have long been regarded as one of the modifiable factors in optimising bone health. Recently, defined as residual or excess hydrogen ion production post-food metabolism [8], dietary acid load (DAL) was proposed to influence bone resorption by reducing urinary calcium excretion. The diet-dependent acid-base balance theory proposes that vegetable and fruits are high in basic elements such as calcium, phosphate, potassium, and magnesium, while animal sources may increase hydrogen ion (H^+^) in blood and may act as acidic food. A Western diet that is high in animal sources and low in vegetable and fruits, can lead to chronic metabolic acidosis and thus causes calcium loss from bone [9,10,11,12,13]. Not much data are available for the Asian population where the projected prevalence of hip fractures is the highest by 2050 [14].

Bone formation and mineralization pathogenesis are highly related to the susceptibility genes such as SPTBN1 (spectrin, β, nonerythrocytic 1) and SOST (sclerosteosis) [15]. However, susceptibility genes, defined as “an increased likelihood of developing a particular disease based on a person’s genetic makeup” [16], do not play the major role in the development of the disease, but rather act as response modifiers to modifiable factors such as diet, physical activity, environment, and medication consumption [17]. Insulin-like growth factor 1 (IGF-1) is a peptide that stimulates bone growth, skeletal maturation and acquisition of bone mass. IGF-1 exerts anabolic effects on trabecular and cortical bone. It increases the synthesis of collagen type 1 and osteocalcin, and stimulates alkaline phosphatase to higher bone mineral density [18] and lower osteoporotic fractures [19,20]. Thus, IGF-1 gene is considered as a potential candidate gene for osteoporotic fracture risk. As genes vary between ethnicities, two recent systematic reviews and meta-analyses by Chen et al. [21] and Gao et al. [22] concluded that one of the common single nucleotide polymorphisms (SNPs) of IGF-1, rs35767, is associated with higher risk of osteoporosis among the Chinese women population.

Although a high animal protein intake is thought to promote acidic homeostasis leading to bone loss and deterioration in skeletal health, several studies reported the opposite findings [8,23]. There were inconsistent findings of whether a high acidic diet might contribute to bone loss. On the other hand, studies support that carriers with IGF-1 rs35767 CC genotype had higher bone mineral densities at the femoral neck, total hip, trochanter and lumbar spine compared with TT and CT genotypes [21] but it remains unknown whether the association between DAL and bone resorption is moderated by gene polymorphism. Therefore, the aim of this study was to examine whether the effects of an alkaline diet on bone resorption among Malaysian Chinese postmenopausal women were moderated by IGF-1 genetic polymorphism. We hypothesize that higher DAL moderated by IGF-1 rs35767 TT and CT genotypes is positively associated with bone resorption among the postmenopausal women.

## 2. Materials and Methods 

### 2.1. Study Design and Participants

This was an analytical cross sectional study conducted in 217 healthy postmenopausal Chinese women that were recruited from the National Council of Senior Citizens Organizations Malaysia (NACSCOM) in Kuala Lumpur and Selangor, Malaysia. The sample size was determined using Gpower software, which gave a power of study of 95%. In this study, two-stage sampling technique was applied. First, simple random sampling was applied to select seven affiliates from the total of 15 affiliates. Second, respondents were recruited based on the inclusion and exclusion criteria. The inclusion criteria were Malaysian; at least 5 years postmenopausal; and not on medication e.g., hormone replacement therapy, aluminium-containing antacids, anticonvulsants, aromatase inhibitors, immunosuppressant, glucocorticoids, proton pump inhibitors, selective serotonin reuptake inhibitors, and thiazolidinediones that could affect bone health within the past one year. Written informed consent was obtained prior to study commencement. Ethical approval was obtained from the Ethics Committee for Research Involving Human Subjects with project reference number was FPSK (FR16) P019.

### 2.2. Sociodemographic and Physical Activity Level

Information on sociodemographic background (age, ethnicity, marital status, education level, occupation, and monthly income) was ascertained using a pre-tested structured questionnaire. Health and medical histories of the participants were self-reported while physical activity level was assessed using Global Physical Activity Questionnaire (GPAQ) [24].

### 2.3. Anthropometric and Blood Pressure Measurements

Body weight and standing height were measured using TANITA digital weighing scale to the nearest 0.1 kg and portable SECA stadiometer to the nearest 0.1 cm, respectively, which allowed the computation of Body Mass Index (BMI). Waist circumference was measured to the nearest 0.1 cm using a non-elastic SECA measuring tape according to standard technique. Blood pressure was measured using a Digital Automatic BP monitor (OMRON HEM-907, Omron, Kyoto, Japan). Participants were asked to sit on a chair with the palm of their hand facing upward and both feet resting on the floor. Measurements were taken twice 10 min apart and an average value was recorded.

### 2.4. Dietary Assessment

Habitual dietary intake of participants for the past one month was assessed by a validated semi-quantitative food frequency questionnaire (FFQ) [25] adapted from the Malaysian Adult Nutrition Survey 2003 [26]. The FFQ consisted of 121 commonly consumed foods by Malaysians. Quantity of food consumed was estimated using a standard serving size based on the ‘Serving size of Malaysian Foods Album’ [27] and the weight of the food item was taken based on the household measurement (Food Portion Sizes of Malaysian Food Album, 2002/2003) [28]. Nutrient and energy intakes were calculated using the Nutritionist Pro™ Diet Analysis (Axxya Systems, Stafford, TX, USA) software while the adequacy of nutrients intake was compared to Recommended Nutrient Intakes for Malaysia (2017) [29]. The conversion of food frequency to the amount of food intake was determined according to the equation below which had been used in previous national surveillance study [30]:

(1) $$\begin{array}{l} Amount\;of\;food\;consumed\;per\;day\;\left(\frac{g}{day}\right)\\ \;=\;frequency\;of\;intake\;\left(conversion\;factor\right)\times \;serving\;size\\ \;\times \;total\;number\;of\;serving\;\times weight\;of\;food\;in\;one\;serving\; \end{array}$$

DAL of the participants was estimated based on the Potential Renal Acid Load (PRAL) equation [31] as below:

(2) $$\begin{array}{cc} PRAL\;\left(mEq/d\right)\; & =\;0.49\;protein\;\left(g/d\right)\\ & +\;0.037\;phosphorus\;\left(mg/d\right)\;-\;0.021\;Potassium\;\left(mg/d\right)\\ & -\;0.026\;Magnesium\;\left(mg/d\right)\;-\;0.013\;Calcium\;\left(mg/d\right) \end{array}$$

### 2.5. Biochemical and Genotyping Measurement

A total of 10 mL fasting venous blood samples was collected from the subjects by qualified phlebotomists. Plasma was separated for biochemical analysis whereas genomic DNA was extracted using a commercial DNA extraction kit (QIAamp DNA Blood Mini Kit Qiagen, Hilden, Germany). The extracted DNA samples were qualified and quantified using agarose gel electrophoresis and Nanophotometer, respectively. The genotyping analysis of IGF-1 gene polymorphism (rs35767 SNP) was analyzed using Agena^®^ MassARRAY platform. SNP analysis was analyzed by Typer Analyzer.

### 2.6. Statistical Analysis

Statistical analysis was performed with SPSS version 22.0 with a significance threshold set at *p* < 0.05. Continuous variables were expressed as mean ± standard deviation while categorical variables as frequencies and percentages. Pearson correlation was used to test the relationship between two variables and *t*-test was employed to determine the significant differences between two means. The Hardy-Weinberg equilibrium for genotypic distribution was evaluated using the Hardy-Weinberg equilibrium exact test. Due to the small number of participants (20 women) who were homozygous of TT genotype and because the bone resorption rate for individuals with TT genotype and CT heterozygote genotype were not significantly different, individuals with TT and CT genotypes were grouped together.

In this study, hierarchical regression was used to test the moderation effect instead of structural equation modeling (SEM) or General Linear Model to test the moderation effect. Although SEM analysis is favorable to conceptualize and evaluate multiple predictors, mediators, or outcomes under an investigation [32], the current study did not have multiple mediators, moderators or outcome. Thus, hierarchical regression analysis may provide more beneficial mathematic information. Previous genetic studies had used hierarchical regression analysis to confirm moderation effect [33,34,35].

A four-step hierarchical regression was performed with the first step (model 1) which investigated the effects of control variables (age, height, years of menopause, calcium intake, serum vitamin D, physical activity level and diabetes) on bone resorption. In the second and third steps (model 2 and model 3), DAL (independent variable) and IGF-1 (moderator) were entered into the models one by one. The last step (model 4) was to test the possible interaction effect of DAL and IGF-1 for predicting the bone resorption. The beta coefficient described the nature of the relationship and the R^2^ indicated the contribution of a set of independent variables on the bone resorption.

Before hierarchical regression, outliers and influential cases were resolved and checked by using residual statistics: Cook’s distance, Leverage, Mahalanobis distance and DFFit. Multicollinearity assumption was measured by tolerance and Variance Inflation Factor (VIF) and Independence of errors assumption was tested by Durbin Watson. Normality, linearity, and homoscedasticity assumption were examined through analysis of residual scatterplots. The four-step hierarchical regression was employed after the model met all the assumptions.

## 3. Results

### 3.1. Background Characteristics of Participants

Table 1 shows the characteristics of participants. The mean age of respondents was more than 65 years while the mean duration of menopause was slightly longer than 15 years. According to the World Health Organization (WHO) classification, more than half of the respondents had normal body weight status. Overweight and obesity were evident, with approximately one in four of the respondents had excess body weight and high body fat percentage. Approximately half of the participants came from a low-income household group with a majority of them had lower secondary education level. Besides, more than one-third failed to achieve the recommended duration of physical activity. Inadequate dietary calcium intake was highly evident whereby less than 10% of the participants achieved the recommended dietary calcium intake. There were 82.4% of the participants fell under the category of either serum 25-hydroxyvitamin D (25(OH)D) deficiency or inadequate, according to the Institute of Medicine guideline [36]. Mean PRAL score was 13.4 ± 19.1, ranging widely from −49.49 to 85.32. Being the surrogate marker of the rate of bone loss, the mean of CTX1 was 0.45 ± 0.17 μg/L, and ranged from 0.05 to 1.2 μg/L.

### 3.2. Bivariate Analysis between Study Variables

The inter-correlation for each variable was presented in Table 2. CTX1 was weakly but significantly correlated with age (*r* = −0.171, *p* < 0.05), with the younger the age, the higher the rate of bone resorption. On the other hand, CTX1 showed no correlation with PRAL score, height, years of menopause, calcium intake, serum vitamin D, and physical activity. There were no significant differences in CTX1 between the IGF-1 genotype and diabetes groups (Table 3).

### 3.3. Moderating Effect of IGF-1

Table 4 shows the four steps hierarchical regression models. Model 1 shows that all control variables (age, height, years of menopause, calcium intake, serum vitamin D, physical activity level and diabetes) were not significantly associated with the CTX1 bone marker. Conversely, there was a significant association between CTX1 and DAL (*F*_(6,207)_ = 2.11, *p* ≤ 0.05, *R*^2^ = 0.079). This model showed that approximately 8% of variance in the rate of bone resorption, as assessed by CTX1 bone marker, was explained by PRAL score. On the other hand, model 3 and model 4 did not show a significant main effect and interaction effect between IGF-1 and CTX1; and between DAL and IGF-1 on CTX1.

## 4. Discussion

The current study showed that mean dietary calcium intake was only 60% of the recommendation [37] with less than 10% of the participants achieved dietary recommendation for calcium. Together with previous findings [7,38,39,40], we further confirmed a consistent habitual low calcium intake among the Asian Chinese postmenopausal population.

A long recognized public health problem among populations residing in subtropical countries [41,42], vitamin D deficiency has emerged as a significant public health issue in tropical countries with almost all year round UV-B radiation being a sufficient wavelength necessary for cutaneous synthesis of vitamin D [43,44,45,46,47], including Malaysia [48,49,50,51]. The finding that less than 20% of the participants met the serum vitamin D recommendation further supports the current evidence of a high prevalence of low serum 25(OH)D level among postmenopausal women [48,52,53]. Vitamin D inadequacy among postmenopausal women may be caused by low sun exposure and lack of outdoor activities, insufficient dietary vitamin D intake and older age [53].

The average estimated PRAL score of our population was slightly higher compared to other studies among the western postmenopausal population [12] suggesting dietary habit of postmenopausal Chinese women in Malaysia are font to an acidic diet. This finding was in contrast with the current belief that Chinese cuisine is high in vegetables and fruits, and lower in animal foods compared to a Western diet [54,55]. More studies are needed to explore this aspect.

The correlation analysis in this study showed that there was a significant and negative correlation between age and rate of bone resorption, suggesting that younger postmenopausal women had a greater rate of bone resorption than older postmenopausal women. It could be explained by the physiological changes of BMD with age. The estrogen deficiency prior to menopause and at the earlier years of menopause induces an imbalance between bone resorption and bone formation. Such imbalance in favor of resorption (uncoupling of bone turnover) results in noticeable bone loss [56] and deterioration of bone architecture. The acceleration of bone resorption usually starts a year before menopause and continues for another 3 years after menopause before a de-accelerating process [57]. The same study also suggested that the bone resorption rate may still be high for 4 to 8 years after menopause.

This study aimed to evaluate the moderating effect of IGF-1 SNP, rs35767, with rate of bone resorption in postmenopausal Chinese women. We hypothesized that CC genotype was the protective genotype and it would potentiate the effect of DAL on reducing the rate of bone resorption. In model 1 (Table 4), it is notable that there were no main effects contributed by the seven control variables (age, height, years of menopausal, calcium intake, serum vitamin D, physical activity level, and diabetes self-report). The results were different from a current systematic review and meta-analysis [58,59]. The variation in genotypes and environmental factors may lead to the development of osteoporosis.

As shown in model 2, higher PRAL value, which indicated a higher diet-dependent proton load, was related to higher rate of bone resorption after adjusting for potential control variables. The result is consistent with several studies that DAL was significantly associated with bone deterioration [10,11,12,13]. This study supports the acid-base theory that bone acts as a primary buffer system for basic components such as calcium, potassium, phosphorus, and magnesium, while the acid component is proton (H^+^) ions. A high protein diet may lead to mild acidosis as it may generate proton (H^+^) ion and increase the acid load. Under a long-term acidic environment, the bone may be called on to act as the buffer to neutralize the acidosis in the body by releasing base mineral [14,60]. However, the relatively small variance could be further delineated with the inclusion of other factors such as renal function, age, gender and immobility in future studies which could have affected the rate of bone formation and resorption [61].

As depicted in Model 3, the pattern of IGF-1 is in line with a previous meta-analysis that higher expressions of allele C contribute to lower bone resorption [21]; however, the moderation effect was not significant in Model 4. As empirical study investigating gene-diet interaction on IGF-1 and dietary acid load were not available, further studies are warranted on this aspect.

One of the strengths of the study was the use of hierarchical regression for testing the relationship between bone resorption and a set of risk factors, and between three genotype groups as moderator and DAL as the variable of interest. Nonetheless, other genetic and biological factors that may affect DAL and IGF-1 on rate of bone resorption were not investigated in this study, which may have contributed to the small variance in explaining the model.

## 5. Conclusions

In conclusion, this study does not support the hypothesis that IGF-1 rs 35767 moderated the effect of DAL and rate of bone resorption. Future research is suggested to expand this study to different ethnicities and to consider other potential genetic or environmental risk factors. Despite its limitation, this is a novel study in determining the effects of SNP and for moderating dietary risk factor on bone loss.

## Figures and Tables

**Table 1:** Characteristics of women (*n* = 217).

Characteristics	Mean ± SD or %
Age (years)	66.69 ± 6.6
Years of menopause (years)	16.07 ± 7.7
Weight (kg)	57.80 ± 9.5
Height (cm)	154.04 ± 5.4
BMI (kg/m^2^)	
Underweight	4.7
Normal	56.5
Overweight	31.8
Obesity	7.0
Body fat percentage (%)	
Underweight	2.8
Normal	54.8
Overweight	28.1
Obese	14.3
Marital status	
Single	9.7
Married	77.4
Divorced	2.8
Others	10.1
Education level (years)	8.0 ± 4.6
Monthly household income (RM)	
<RM2300	44.2
RM2300–RM5599	36.9
≥RM5600	18.9
Physical activity (MET-min/week)	1034.8 ± 937.9
Below recommendation	38.1
Meeting recommendation	61.9
Calcium intake (mg/day)	617.9 ± 278.6
Below recommendation	93.1
Meeting recommendation	6.9
Presemce of diabetes mellitus	
Yes	12.4
No	87.6
Serum vitamin D (ug)	37.5 ± 14.3
Deficiency	33.6
Inadequate	48.8
Adequate	17.5
PRAL score (mEq/day)	13.4 ± 19.1
25%ile	2.0426
50%ile	12.1016
75%ile	24.9106
IGF-1 (rs35767) (genotype)	
CC	47.2
CT	43.5
TT	9.3
CTX1 (μg/L)	0.45 ± 0.17
25%ile	0.3125
50%ile	0.41
75%ile	0.57

Note: BMI, body mass index; CTX1, serum collagen type 1 cross-linked C-telopeptide; 1 USD was equivalent to 4.29 MYR (Ringgit Malaysia) at time of data collection (June 2017).

**Table 2:** Bivariate Pearson correlation coefficients for rate of bone resorption (CTX1), PRAL score and controls.

Variable	1	2	3	4	5	6	7	8
1. CTX1	1	0.097	−0.171 *	0.103	−0.109	−0.079	−0.084	0.065
2. PRAL score		1	0.096	−0.023	−0.146 *	0.059	0.118	−0.067
3. Age			1	0.246 **	0.761 **	0.022	0.006	−0.104
4. Height				1	−0.145 *	0.077	−0.067	0.032
5. Years of menopause					1	0.083	−0.017	−0.091
6. Calcium intake						1	0.085	0.053
7. Serum vitamin D							1	0.049
8. Physical activity								1

* *p* < 0.05, ** *p* < 0.01. Note: CTX1: serum collagen type 1 cross-linked C-telopeptide, PRAL: potential renal acid load.

**Table 3:** Independent-sample t test between IGF-1 genotypes, diabetes and rate of bone resorption (CTX1).

Variable	*n*	Mean	SD	*t* Value	Sig-*t*
IGF-1 genotype				0.986	0.325
CC	101	0.432	0.196		
TT and CT	113	0.457	0.180		
Presence of diabetes mellitus				−1.274	0.204
Yes	27	0.403	0.165		
No	189	0.452	0.192		

**Table 4:** Hierarchical regression analysis of factors predicting bone loss (CTX1).

Predictors	Beta	*p*	R^2^	F	Sig-F
Model 1 (control variables)			0.055	1.65	0.123
Age	−0.192	0.08			
Height	0.042	0.554			
Years of menopause	0.053	0.624			
Calcium intake	−0.083	0.237			
Serum vitamin D	−0.084	0.235			
Physical activity	0.055	0.43			
Presence of diabetes mellitus (Yes = 1, No = 0)	0.05	0.484			
Model 2 (control variables + IV of interest)			0.079	2.110	0.036 *
Age	−0.186	0.089			
Height	0.031	0.665			
Years of menopause	0.027	0.806			
Calcium intake	−0.085	0.223			
Serum vitamin D	−0.112	0.113			
Physical activity	0.06	0.385			
Presence of diabetes mellitus (Yes = 1, No = 0)	0.067	0.347			
PRAL score	0.152	0.031 *			
Model 3 (control variables + IV + moderator)			0.079	1.858	0.06
Age	−0.197	0.074			
Height	0.028	0.696			
Years of menopause	0.032	0.769			
Calcium intake	−0.066	0.352			
Serum vitamin D	−0.111	0.118			
Physical activity	0.043	0.537			
Presence of Diabetes Mellitus (Yes = 1, No = 0)	0.056	0.432			
PRAL score	0.147	0.040 *			
IGF-1 rs35767 (CC = 0, TT + CT = 1)	0.061	0.384			
Model 4 (control variables + IV + moderator + moderator × IV)			0.083	1.737	0.075
Age	−0.193	0.082			
Height	0.035	0.632			
Years of menopause	0.027	0.807			
Calcium intake	−0.072	0.311			
Serum vitamin D	−0.112	0.116			
Physical activity	0.045	0.517			
Presence of Diabetes Mellitus	0.054	0.452			
PRAL score	0.213	0.048 *			
IGF-1 rs35767 (CC = 0, TT + CT = 1)	0.102	0.237			
PRAL*IGF1 rs35767	−0.097	0.413			

Note: * *p* < 0.05, CTX1, serum collagen type 1 cross-linked C-telopeptide; PRAL, potential renal acid; IGF-1, insulin-like growth factor-1.
